# Long-Term Clinical Benefit in *EGFR*-Mutant Lung Adenocarcinoma With Local Squamous Cell Carcinoma Transformation After EGFR TKI Resistance: A Case Report

**DOI:** 10.3389/fonc.2022.883367

**Published:** 2022-05-26

**Authors:** Junru Ye, Yutong Ma, Qiuxiang Ou, Junrong Yan, Bin Ye, Yuping Li

**Affiliations:** ^1^ Department of Pulmonary and Critical Care Medicine, The First Affiliated Hospital of Wenzhou Medical University, Wenzhou, China; ^2^ Geneseeq Research Institute, Nanjing Geneseeq Technology Inc., Nanjing, China; ^3^ Medical Department, Nanjing Geneseeq Technology Inc., Nanjing, China

**Keywords:** adenocarcinoma, squamous cell carcinoma, PD-L1, EGFR TKI, case report

## Abstract

The histological transformation from adenocarcinoma (ADC) to squamous cell carcinoma (SCC) is rare but recurrently occurs post TKI treatment in *EGFR-*mutated non-small cell lung cancer patients with a very limited number of clinical cases published. The outcome of patients after SCC onset is poor as no established treatment guidelines were available. Here we report a case who was initially diagnosed with lung ADC with *EGFR* L858R driver mutation and demonstrated a partial response (PR) to gefitinib for 27 months before disease progression. The rapidly progressive lung metastatic lesions were determined as an SCC histology with positive PD-L1 expression. Besides *EGFR* L858R, the metastatic SCC harbored the amplification of *CD274* and *PDCD1LG2* detected by targeted next-generation sequencing (NGS), which encode PD-L1 and PD-L2, respectively. The disease remained stable on the combination therapy of pembrolizumab plus chemotherapy for eight months until the primary ADC lesion progressed. After the failure of progressed primary ADC lesion with radiotherapy and immunotherapy, systemic ADC metastases were developed in multiple locations including kidney, liver, and chest wall with *EGFR* L858R mutation but negative PD-L1 expression. The patient then received the combination therapy of bevacizumab plus chemotherapy and the disease remained stable for five months. Since August 2021, afatinib has been administrated which led to a PR and the disease has remained stable up till present. This study demonstrated a primary lung ADC who developed systemic ADC metastases and local SCC transformation with distinct molecular features. The patient has achieved long-term clinical benefit upon multiple lines of chemotherapy and immunotherapy, which provided valuable insight into the treatment of advanced SCC-transformed lung ADC patients.

## Introduction

The most important driver gene in non-small cell lung cancer (NSCLC) is *EGFR* and approximately 50% of Chinese NSCLC patients carry *EGFR* mutations, among which, L858R and exon 19 deletion are the two most common alterations (1). Patients with activating *EGFR* mutations usually showed optimal responses to EGFR tyrosine kinase inhibitors (TKIs). However, the acquired mutations during TKI therapy would eventually develop resistance, such as *EGFR* T790M and *MET* amplification (2).

Besides the acquired secondary alterations, the change of histologic phenotype could also lead to the TKI resistance. Adenocarcinoma (ADC) and squamous cell carcinoma (SCC) are the two major subtypes of NSCLC and due to the heterogenicity, about 4% to 9% of tumors are mixed with both ADC and SCC, even in the same lesion (3). Immunochemistry (IHC) examination is a clinical approach to distinguish the two histology with specific biomarkers such as thyroid transcription factor (TTF-1), NapsinA, CK5/6, and P63. In addition, *EGFR* oncogenic mutations and *ALK/RET* fusions are mostly detected in ADCs (4). The transdifferentiation from ADC to SCC was supported by both *in vivo* and clinical evidence. In the genetically engineered mouse model, the loss of *LKB1* was proven to be a mechanism of triggering the ADC-SCC transformation (5). In real-world cases, the prognosis of ADC-to-SCC transformed NSCLC patients was extremely poor and a 3.5-month median overall survival (OS) after SCC onset was reported in a systematic literature review (6).

Herein we presented a rare case of lung ADC with *EGFR* L858R driver mutation developing local SCC transformation after first-line gefitinib treatment followed by systemic ADC metastases. Based on repeated pathological examinations and targeted next-generation sequencing (NGS), the rapidly progressive disease was under control and long-term clinical benefits were achieved. We present the following case in accordance with the CARE reporting checklist.

## Case Presentation

**Figure 1:**
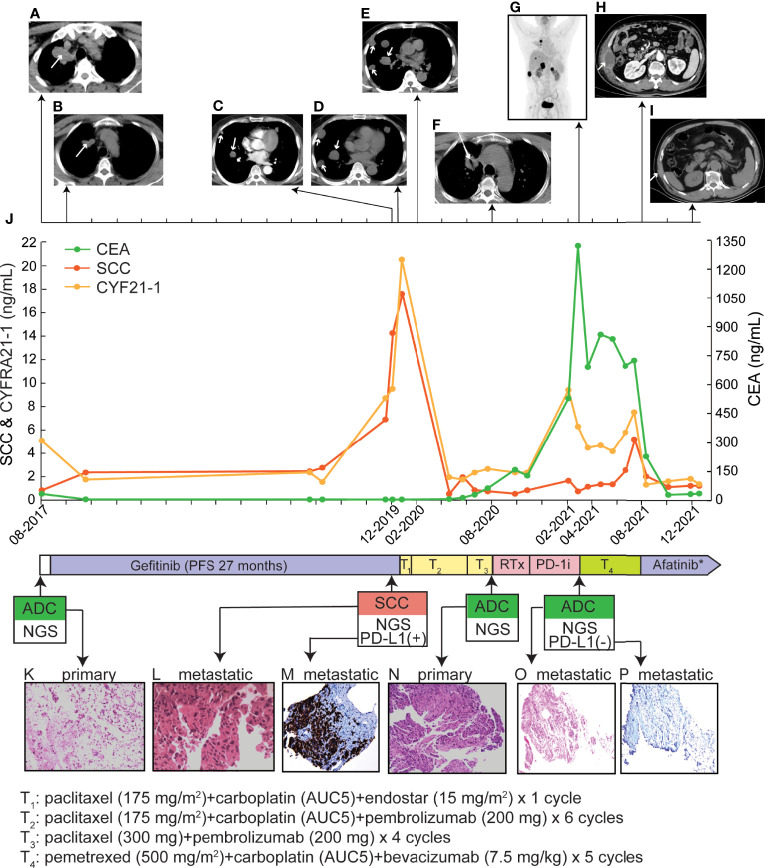
Treatment history and clinical information of the presented case. **(A–I)** PET/CT images of primary and metastatic lesions were found at different time points as shown by the time scale. **(J)** Multiple serum tests of SCC antigen, CYFRA21-1, and CEA were performed. **(K**, **L**, **N**, **O)** Pathological examination of primary and metastatic lesion biopsy. The magnificence of the H&E staining images was 100x. **(M**, **P)** The IHC examination of PD-L1 expression. The tumor proportion score of the metastatic SCC lesion and the chest wall lesion were > 50% and < 1%, respectively. RTx, radiotherapy; *, during the first month of afatinib treatment, anlotinib was co-administrated, which was discontinued due to side effects.

A 61-year-old non-smoking Chinese male visited our hospital in August 2017, because of a persistent cough for over one month. He had hypertension for two years, taking lercanidipine tablets for blood pressure control, without any medical history of diabetes, pulmonary tuberculosis, or drug allergy. Positron emission tomography (PET) and computed tomography (CT) examinations revealed the presence of a lesion in the right upper lobe (**Figure 1A**) and multiple lymph nodes metastases at the right hilum of the lung, right mediastinum, the right-side clavicle, and pleural effusion in the right side. The percutaneous biopsy and immunohistochemistry (IHC) analysis revealed a poorly differentiated adenocarcinoma (ADC, **Figure 1K**) with positive expression of Napsin A, thyroid transcription factor-1 (TTF-1), and negative CK5/6. The *EGFR* L858R mutation was detected (**Table 1**) by the capture-based hybrid next-generation sequencing (NGS, GeneseeqPrime™) targeting 425 cancer-related genes (**Supplementary Table S1**). He was primarily diagnosed with a stage IV ADC of the right lung (T2N3M1a) and received gefitinib as the first-line treatment. Partial response (PR) was achieved after one month (**Figure 1B**) and progression-free survival (PFS) was 27 months for the first-line gefitinib treatment. During the treatment of gefitinib, the patient experienced grade 2 rash, which was symptomatically treated.

**Table 1:** NGS-detected alterations in multiple lesion sites.

	Primary Lung (ADC) 2017.08	Metastatic Lung (SCC)2019.12	Primary Lung (ADC) 2020.08	Metastatic Chest Wall (ADC) 2021.03
*EGFR*: L858R	20%	14.50%	33.40%	24.80%
*TP53*: E56*	28.12%	17.60%	27.30%	21.10%
*CDKN2A~CDKN2B* upstream fusion	21.95%	15.70%	19.40%	30.90%
*PIK3CA*: E545K	9.68%		12.70%	11.10%
*EGFR*: S306L	34.25%		31.40%	26.80%
*PGR*: E390K		11.90%	11.70%	7.20%
*MET*: amp		4-fold		
*CD274*: amp		6.5-fold		
*PDCD1LG2*: amp		6.8-fold		
*TERT*: amp		2.5-fold		
*EGFR:* S306L		14.90%		
*GRM3*: K448R		9.90%		
*YAP1*: R289W		10.30%		
*DOT1L*: P12L	6.17%			
*TERT*: P219L	4.23%			
*MET*: V1066Gfs*4			6.60%	7.20%

amp, amplification; ADC, adenocarcinoma; SCC, squamous cell carcinoma. *, termination codon.

In December 2019, multiple metastatic lesions were found in both lungs and rapidly progressed. As shown in **Figures 1C, D**, the two CT images taken with an interval of 19 days (2019.12.06 and 2019.12.25) showed over two times size increase on multiple metastatic sites. As the primary tumor remained stable, a re-biopsy of the metastatic lesion was performed. However, the pathological examinations suggested a histology change to squamous cell carcinoma (SCC, **Figure 1L**) with positive expression of CK5/6 and P63 but negative for NapsinA and TTF-1. The SCC antigen and CYFRA21-1 levels in serum were significantly increased compared to the baseline tests (**Figure 1J**). He firstly received one cycle of paclitaxel (175 mg/m^2^), carboplatin (AUC5) plus endostar (15 mg/m^2^). Then, the IHC test showed a positive PD-L1 expression with a tumor proportion score (TPS) of over 50% (**Figure 1M**). The L858R mutation of *EGFR* was maintained in the metastatic SCC and companied by the amplification of *MET*, *CD274*, and *PDCD1LG2* (**Table 1**). Thus, a combination of paclitaxel (175 mg/m^2^), carboplatin (AUC5) plus pembrolizumab (200 mg) was administrated. The follow-up examination after one cycle of combined treatment showed that the rapidly progressed metastatic lesions had been under control (**Figure 1E**). Then, partial response was achieved after an additional 5 cycles of this combined therapy and another four cycles of paclitaxel (300 mg) plus pembrolizumab (200 mg) were performed as maintained treatment. The disease remained stable until August 2020 and the patient tolerated well with current treatment.

Due to the progression of the primary tumor in August 2020, a second biopsy for the primary site was performed (**Figure 1F**), whose pathologic histology was still ADC (**Figure 1N**) with dramatic decreases in the levels of SCC antigen and CYFRA21-1 in serum (**Figure 1J**). Compared to the mutational profile of the metastatic SCC lesion, the progressed ADC maintained the oncogenic *EGFR* (L858R) mutation. However, the amplification of *MET*, *CD274*, and *PDCD1LG2* was not detected (**Table 1**). Whereas no genetic alterations were detected in the circulating tumor DNA by targeted NGS which suggested a very low level of tumor mutational burden. After a total of 3600cGy radiotherapy targeting the progressed primary lesion (12 times in 4 weeks), the patient continued pembrolizumab (200 mg) treatment until the March of 2021.

Multiple metastatic sites were found by repeat PET-CT including kidney, liver, and chest wall (**Figure 1G**) with a dramatic increase in carcinoembryonic antigen (CEA) level (**Figure 1J**). The metastatic chest wall lesion was pathologically identified as ADC (**Figure 1O**) with the same genetic alterations as the progressed ADC (**Table 1**). Due to the clearance of the gene amplification of *CD274* and *PDCD1LG2*, the IHC test of PD-L1 was negative for the chest wall lesion (**Figure 1P**). A combination treatment of pemetrexed (500 mg/m^2^), carboplatin (AUC5), and bevacizumab (7.5 mg/kg) led to a stable disease for five months until August 2021. With the progression on the metastatic chest wall lesion (**Figure 1H**), afatinib (40 mg/d) and anlotinib (10 mg/d) were administrated to the patient, but anolotinib was discontinued after one month because of severe side effects, including hemoptysis and tooth loss. Afatinib monotherapy relieved chest pain and led to a PR of the metastatic chest wall lesion as shown by the CT scan in December 2021 (**Figure 1I**). Until the last follow-up in January 2022, the PFS upon afatinib treatment reached five months and the treatment is ongoing.

## Discussion

In this case report, we presented an NSCLC patient experiencing a histologic transformation of ADC to SCC in the local metastatic lesions. The primary tumor was diagnosed as ADC with *EGFR* L858R driver mutation and the efficacy of first-line gefitinib treatment was excellent with a 27-month PFS. While a histological transformation to SCC occurred in the local metastatic lesion. The histological transformation is a known resistant mechanism of EGFR-TKI treatment in NSCLC patients. About 3% to 14% of patients trans-differentiated to small-cell lung cancer (SCLC) which mediated the resistance to EGFR-TKI treatment (7, 8). In addition, *EGFR-*mutant patients with co-alterations of *RB1* and *TP53* experienced a higher risk of SCLC transformation (9). However, we didn’t detect any *RB1* mutations during multiple sampling, though *RB1* gene was covered by the targeted NGS panel used in this case (**Supplementary Table S1**). Thus, the molecular traits of patients experiencing ADC-to-SCC transformation might be different from those transformed to SCLC, which remained to be well characterized (10). In the engineered mouse model (*Kras^G12D^/Lkb1^L/L^*), *LKB1* was proven to play a critical role in ADC-to-SCC transdifferentiation (5). Due to the limited number of reported cases, the clinical evidence was poorly investigated.

As the transformation from *EGFR-*mutated ADC to SCC is relatively rare, no established treatment guidelines were available in this scenario. According to the published cases, the transformation from ADC to SCC could occur post first-, second-, and third-generation TKIs including gefitinib, erlotinib, afatinib, and osimertinib (6). *EGFR* T790M is a recurrent mutation detected in transformed SCC tumors and osimertinib was often chosen as the subsequent therapy but the clinical benefits were not durable (11–14). Roca et al. reported a 3.5-month median OS of *EGFR-*mutant NSCLC patients after SCC transformation in a pooled analysis (6). In the presented case, the combined chemotherapy and immune checkpoint inhibitor (ICI) slowed down the rapid progression of the metastatic SCC tumors with an eight-month stable disease. The amplification of *CD274* and *PDCD1LG2* were detected by targeted NGS in the metastatic SCC tumors which encode PD-L1 and PD-L2, respectively. Thus, the positive expression of PD-L1/2 could explain the good response to pembrolizumab. In addition, previous studies have demonstrated the diverse responses to ICI in NSCLC patients with different *EGFR* alterations by affecting tumor immune microenvironments (15–17). While preclinical experiments showed that EGFR TKIs could improve ICI efficacy by triggering the changes in tumor immune microenvironments, which implicated the combination with ICI in clinical practice (18, 19). However, in this case, the progressed primary ADC lesion and the systemic metastatic tumors lost the amplification of *CD274* and *PDCD1LG2*, which might trigger the resistance to immunotherapy. Besides, other resistant mechanisms have been reported, including the selection and accumulation of tumor subclones that escaped from the immune system, the activation of other compensatory inhibitory signaling pathways, and the exhaustion of T cells (20–22).

With the application of large-panel NGS analysis, we were not only able to identify oncogenic driver mutations, *EGFR* L858R in this case, but also other concurrent alterations that might assist treatment decision-making and predict therapeutic responses. For instance, a *TP53* nonsense mutation was carried in both primary and all metastatic tumors. Concurrent *TP53* mutation in *EGFR-*mutant lung cancer patients was associated with a poor prognosis with TKI treatments (23) but in this case, the patient derived benefit from gefitinib for 27 months. Notably, a four-fold amplification of *MET* gene was detected in the metastatic SCC tumor, which was also a known resistant mechanism to EGFR TKIs. Thus, beyond the histological transformation, *MET* amplification might also trigger the development of local metastases. Another genomic alteration maintained in all samples was a gene fusion where exon 1 of *CDKN2A* was translocated to the upstream intergenic region of *CDKN2B*. But whether this DNA-level alteration eventually cause transcription- and protein-level changes was unknown. Previous studies reported the association between *CDKN2A* deletion and poor clinical prognosis in lung cancer (24, 25).

This study has several limitations. First, whether the primary tumor underwent SCC transformation remained unclear because a histology examination was lacking when SCC was identified but the primary remained stable. Secondly, the original pathological examinations were performed based on percutaneous biopsies. Hence, the *de novo* primary tumor might be a mix of ADC and SCC due to intratumor heterogeneity and the SCC subclone became dominant and migrated during the first-line gefitinib treatment. However, as the NGS results revealed that the molecular profile of the SCC was quite different from that of the primary ADC, and the metastatic ADC lesions at chest wall maintained the majority of alterations detected in the primary ADC, we preferred the hypothesis that the trans-differentiation occurred during the TKI treatment and migrated to local sites. Thirdly, from this single case, we cannot conclude whether the chemotherapy plus immune checkpoint inhibitors could be widely used in other SCC-transformed EGFR-mutant NSCLC cases. Comprehensive studies of the mechanisms underlying ADC to SCC in large cohorts are warranted.

In summary, we presented a lung cancer patient experiencing an ADC-to-SCC transformation in the local metastatic lesions post 27-month gefitinib treatment. The metastatic SCC responded well to the combined chemotherapy and pembrolizumab with positive PD-L1 expression. As the outcomes of patients with SCC-transformed advanced lung cancer are usually poor, the strategies of the multiple lines of treatments in the presented case are of great value in clinical practice, which successfully stopped the rapid progression of SCC tumors and achieved durable benefits after the development of systemic ADC metastases.

## Data Availability Statement

The original contributions presented in the study are included in the article/**Supplementary Material**. Further inquiries can be directed to the corresponding author.

## Ethics Statement

The studies involving human participants were reviewed and approved by the committee of the first affiliated Hospital of Wenzhou Medical University. The patients/participants provided their written informed consent to participate in this study.

## Author Contributions

JYe: Conceptualization, Data curation, Writing-original writing; YM: Formal analysis, Visualization, Writing-original writing; QO: Validation, Writing-review and editing; JYa: Formal analysis, Resources; BY: Methodology, Validation; YL: Project administration; Supervision, Resources. All authors contributed to the article and approved the submitted version.

## Conflict of Interest

YM, QO, JYa, and BY are current employees of Nanjing Geneseeq Technology Inc.

The remaining authors declare that the research was conducted in the absence of any commercial or financial relationships that could be construed as a potential conflict of interest.

## Publisher’s Note

All claims expressed in this article are solely those of the authors and do not necessarily represent those of their affiliated organizations, or those of the publisher, the editors and the reviewers. Any product that may be evaluated in this article, or claim that may be made by its manufacturer, is not guaranteed or endorsed by the publisher.

## Abbreviations

ADC, adenocarcinoma; SCC, squamous cell carcinoma; NSCLC, non-small cell lung cancer; TKI, tyrosine kinase inhibitor; IHC, immunochemistry; TTF-1, thyroid transcription factor-1; OS, overall survival; PET, positron emission tomography; NGS, next-generation sequencing; PR, partial response; PFS, progression-free survival; TPS, tumor proportion score; SCLC, small-cell lung cancer; CEA, carcinoembryonic antigen; ICI, immune checkpoint inhibitor. Note: RTx, radiotherapy; *, during the first month of afatinib treatment, anlotinib was co-administrated, which was discontinued due to side effects.
